# LGBTQ+ Students’ Peer Victimization and Mental Health before and during the COVID-19 Pandemic

**DOI:** 10.3390/ijerph191811537

**Published:** 2022-09-13

**Authors:** Erin K. Gill, Mollie T. McQuillan

**Affiliations:** Department of Educational Leadership and Policy Analysis, University of Wisconsin-Madison, Madison, WI 53706, USA

**Keywords:** COVID-19, LGBTQ+, adolescence, victimization, anxiety, suicide, gender, sexual orientation, school climate

## Abstract

Given the well-established health disparities between lesbian, gay, bisexual, transgender, queer, and gender-expansive (LGBTQ+) and cisgender, straight youth, scholars predicted the COVID-19 pandemic would disproportionately impact LGBTQ+ students. Yet, few studies have described changes in LGBTQ+ students’ school experiences and well-being during the pandemic. Using survey data from 40,904 middle and high school students, we examined changes from before (2018) to during (2021) the pandemic in peer victimization, anxiety, and suicide attempts. We conducted bivariate and multivariate regression analyses to assess changes between the two time points among LGBTQ+ and cisgender, straight students. We found LGBTQ+ students experienced more peer victimization, anxiety, and suicide attempts at both points, before and during the pandemic, than cisgender, straight youth. However, LGBTQ+ students reported increased anxiety, decreased peer victimization, and decreased suicide attempts in 2021, during the pandemic, compared to pre-pandemic 2018 reports. These findings suggest pandemic-related school disruptions may have provided LGBTQ+ students some relief from the harmful effects of poor, in-person school climates.

## 1. Introduction

The COVID-19 pandemic disrupted K-12 education, exacerbating long-standing academic, social, and health disparities among historically marginalized groups of students [1,2,3,4,5]. However, it remains unclear what effect COVID-19-related disruptions to the school environment had on lesbian, gay, bisexual, transgender, queer, and gender-expansive (LGBTQ+) youth. The pre-pandemic literature concerning LGBTQ+ students’ school experiences suggests that COVID-19-related school disruptions could have both beneficial and harmful consequences for youth. When schools closed their doors to in-person learning and shifted to virtual learning in March of 2020, families with school-aged children faced multiple obstacles. Caregivers lost jobs, moved to remote work, scrambled to find childcare, learned about new work- and school-related technology, supported their children with online learning, and lost access to many social supports that schools provide for families. Reports of increased stress and anxiety in the United States since the start of the COVID-19 pandemic reflect these realities [6]. Because of the well-established academic and health disparities between cisgender, straight and LGBTQ+ youth before the pandemic and the added stress COVID-19 posed to families with school-aged children, many scholars raised concerns about the health and safety of LGBTQ+ students [7,8,9,10]. These scholars suggested that school closures could further increase exposure to family-based social stressors as students lost access to LGBTQ+-inclusive supports in schools [7,9,10].

To that end, school closures related to the COVID-19 pandemic could have also provided LGBTQ+ students sanctuary from harmful school environments. If students could still access support, the change to online learning could shield students from the prevalent, harmful social interactions. Prior to the COVID-19 pandemic, LGBTQ+ students faced greater identity-based social stressors in schools and at home on average compared to their peers, which put LGBTQ+ youth at risk of poor academic and health outcomes [11,12,13,14,15,16,17,18,19,20,21]. Yet, LGBTQ+ youth in environments with greater structural and social support do not necessarily report poor academic or health outcomes [18,22,23,24,25], indicating the importance of school and family contexts on these individual-level outcomes. Students might have also been spared from harmful school environments with high levels of bullying and discrimination because of COVID-19-related school disruptions and virtual learning spaces.

A more nuanced understanding of the impact of COVID-19 on LGBTQ+ students aligns with calls from a growing number of scholars who have advocated for educational and health researchers to move beyond dominant narratives of LGBTQ+ youth as victims of bullying [26,27,28,29,30,31]. These scholars recommend focusing on how schools maintain cisheteronormative systems, while also describing the agentic power of LGBTQ+ youth. For instance, Brockenbrough [26] and other scholars [28] using a queer of color critique lens advocate for the field to focus on systemic oppression through racist and cisheteronormative educational institutions. Payne and Smith [29] similarly argue that efforts to reduce risk by intervening in anti-LGBTQ+ bias in schools do not account for the many dimensions of institutionalized power that marginalize LGBTQ+ youth. Gilbert et al. [27] emphasize research that resists deficit-based narratives and highlight new possibilities for students within schools. Rather than focusing on the individual, this paper aims to center the influence of systemic disruptions on the well-being of youth. The COVID-19 disruptions provide an opportunity to examine how in-school forces influence the safety and well-being of LGBTQ+ youth across multiple domains. Thus, this paper explores changes in students’ reports of victimization, anxiety, and suicide attempts during the COVID-19 pandemic and whether LGBTQ+ students reported different trends across these constructs compared to cisgender, straight students.

### 1.1. Schools as Sites of Harm and Support for LGBTQ+ Youth

Prior to the pandemic, many LGBTQ+ youth experienced marginalizing school environments that negatively influenced their well-being. LGBTQ+ youth face higher incidents of harassment, bullying, and victimization in schools than their heterosexual and cisgender peers [13,15,16,19,32], and this rate has remained relatively stable since 2015 [33]. In a 2019 national survey of LGBTQ+ students, more than 80% of LGBTQ+ students reported experiencing harassment or assault at school [22]. Using the Dane County Youth Assessment (DCYA), a survey of middle and high school students, Robinson and Espelage [20] found that LGBTQ+ students reported less school belonging and greater suicide attempts, victimization, and unexcused absences.

Harassment and the related health risks, such as suicide, can also be exacerbated by direct harassment or a lack of intervention by adults in schools. For instance, several scholars find that educators do not consistently intervene in LGBTQ+ bias-based bullying [34,35,36]. Additionally, more than half of LGBTQ+ students reported hearing school staff make homophobic comments, and two-thirds reported hearing school staff make negative remarks about students’ gender expression [22]. McQuillan and Mayo [37] suggest educational practitioners contribute to ongoing bias and bullying in schools by directly bullying LGBTQ+ youth and preventing staff from learning how to better support students.

Exposure to identity-based stigma, victimization, or discrimination puts LGBTQ+ students at greater risk than their cisgender and heterosexual peers of experiencing poor academic achievement and mental health problems [11,12,20,32,38]. Several pre-pandemic studies suggest suicide attempts among the general U.S. population has remained relatively unchanged in the last decade [39,40,41], although the rates among adolescents have increased according to the Centers for Disease Control [39,42,43,44]. Although suicide rates have been decreasing among LGB youth, Raifman et al. [45] indicate that LGB youth remain 3 times more likely to attempt suicide. The lack of longitudinal data makes similar trend analysis of suicide attempts in transgender youth difficult. Still, Toomey et al. [46] report transgender youth are 3.5 times more likely to attempt suicide than cisgender youth. Using the DCYA data, Robinson and Espelage [32] found bullying to partially explain mental health risks among LGBTQ+ youth, such as suicide attempts, but are only part of the contributing factors. As such, our consideration of the effect of systemic disruptions during the COVID-19 pandemic includes an assessment of changing suicide attempts rates.

Although schools can be a stressful location for LGBTQ+ youth, they are also sites where youth can explore different aspects of their identity and develop identity-based leadership skills. Schools can provide access to important supportive relationships with peers and adults, as well as to resources that affirm their gender or sexual identity [27]. This support contributes positively to students’ well-being [17,22,47,48,49,50]. Connecting with supportive peers and adults at school can facilitate greater school belonging and other positive school experiences compared to youth without supportive social support in school [17,22,50]. Social support from peers can also mitigate the harmful effects of family rejection on LGBTQ+ youth’s mental health [51]. Educators may support LGBTQ+ youth by discussing LGBTQ+ issues, supporting leadership skills, and connecting students to affirming resources. LGBTQ+ students in schools with supportive school personnel experience a greater sense of belonging, less victimization, higher self-esteem, and lower levels of depression than those without supportive adults in schools [17,22,50]. Social support and safe spaces may have been even more important for LGBTQ+ youth during the pandemic [9,10,52].

In addition to supportive peers and adults, access to LGBTQ+-inclusive school resources promotes LGBTQ+ students’ mental health and safety in schools [27,53,54,55]. For instance, Gender and Sexuality Alliances (GSAs) provide safe spaces for LGBTQ+ youth in schools where they can explore their identities [56,57,58,59]. LGBTQ+ students in schools with GSAs report feeling safer and having lower rates of mental health problems than those without GSAs [60,61,62]. School-based health centers also provide critical access to mental health services. LGBTQ+ students are more likely to utilize school-based health services than other mental health services, and school-based health services are associated with lower mental health distress among LGBTQ+ youth [63,64]. Additionally, LGBTQ+ students in schools with protective policies, LGBTQ+-inclusive curricula, and practices report better school experiences and well-being than students without protective and inclusive supports [49,55,65,66,67,68,69]. The COVID-19 pandemic may have restricted LGBTQ+ students’ access to these affirming resources and exacerbated existing mental health issues.

### 1.2. The COVID-19 Pandemic and LGBTQ+ Youth

School closures moved students out of schools and into home environments that may have posed serious risks to LGBTQ+ students’ health and safety. Coming out to family and friends, as well as receiving their support has been important to LGBTQ+ individuals’ health [70,71]. LGBTQ+ youth who conceal their identity [72,73] or are rejected from their families [21] experience greater mental health problems (i.e., distress, depression, anxiety, disordered eating, suicide). During the pandemic, only one-third of LGBTQ+ youth reported that they found their home affirming to their gender or sexual identity [74]. Even more concerning, LGBTQ+ youth report higher rates of sexual, psychological, and physical abuse from their families than their cisgender, heterosexual peers [75,76]. LGBTQ+ youth who have yet to disclose their gender or sexual identity, or who are “out” but lack support from their families, face additional stressors being at home [3,9,10,77]. The COVID-19 pandemic may have exacerbated mental health problems among LGBTQ+ youth as they spent extended time at home, especially for youth among unsupportive families.

Some early evidence suggests the COVID-19 pandemic put LGBTQ+ individuals’ at risk of experiencing emotional distress. Overall, LGBTQ+ adults experienced increased stress, anxiety, depression, and decreased quality of life during the pandemic [78,79,80]. Although studies exploring these relationships among K-12 LGBTQ+ students remain scarce, at least one study found LGBTQ+ college students who reported increased victimization during the COVD-19 pandemic had four times the odds of higher levels of psychological distress [52]. Qualitative studies indicate K-12 LGBTQ+ youth expressed pandemic-related concerns (1) for their mental health due to social isolation and a lack of structure, (2) being at home with unsupportive family members, and (3) loss of access to LGBTQ+-inclusive spaces and resources in schools [77]. Compared to 30.3% of heterosexual youth, 63.8% of lesbian, gay, and bisexual youth reported that their “mental health was most of the time or always not good during the COVID-19 pandemic” [43]. Initial research indicates the COVID-19 pandemic exacerbated mental health concerns among LGBTQ+ individuals.

### 1.3. Current Study

The current study expands upon the initial pandemic literature to explore changes in peer victimization, anxiety, and suicide attempts using survey data before and during the COVID-19 pandemic. We also examine how students’ sexual orientation and gender moderated changes in peer victimization, anxiety, and suicide attempts from 2018 to 2021. We hypothesized that (1) we would find significant differences in victimization, anxiety, and suicide attempts between LGBTQ+ and straight, cisgender students before and during the pandemic, and (2) that LGBTQ+ status would moderate the changes at the beginning of the COVID-19 pandemic in peer victimization, anxiety, and suicide attempts.

## 2. Materials and Methods

### 2.1. Data

Data for this study comes from the 2018 and 2021 Dane County Youth Assessment (DCYA), a survey study conducted by the Public Health Madison Dane County and Dane County Youth Commission. In 2018 and 2021, 7th–12th grade students in Dane County, Wisconsin participated in the DCYA (N = 44,288). The DCYA is conducted every three years and monitors protective and risk behaviors among youth [81,82]. Student participation took place in schools in 2018 and participants took the survey both in schools and at home online in 2021. The survey is voluntary, anonymous, and parents may opt their child out of participating.

Most school districts in Dane County attempt to survey all 7th–12th graders in their district, but the two largest school districts survey 50% of their students. The 2018 survey was administered between January and March 2018 to students across 17 public school districts and one private school. Among the districts that aim to survey all students (16 out of 18), most districts captured over 90% of students. The 2021 survey was administered between January and April of 2021 to students from 19 public school districts and one private high school. Among the districts that aim to survey all students (18 out of 20), most districts captured over 80% of students. Both the 2018 and 2021 DCYA data are weighted (based on school census and demographic data) to ensure representation within Dane County’s 7th–12th grade population. 

The analytic sample consists of 40,904 public middle and high school students. Our study included constructs measured by both the middle and high school DCYA questionnaires. Thus, we used peer victimization, anxiety, and suicide attempts as our main dependent variables. We did not include depression because only the high school questionnaire collected data on this construct. We excluded participants with missing observations in the main identity variables: sexual orientation, gender identity, transgender identity, race, and year variables (7.65%). Additionally, we mean-replaced missing values of main outcome variables of interest stratified by sexual orientation and year (>10%). We present the demographic characteristics of participants in the analytic sample in Table 1.

### 2.2. Measures

#### 2.2.1. Year

The Dane County Youth Assessment (DCYA) collects data every three years. We used two-time points in our analyses. The binary year variable (0 = 2018, 1 = 2021) captures whether the data were collected pre-pandemic (2018) or mid-pandemic (2021), and serves as a main variable of interest. 

#### 2.2.2. LGBTQ+ Status

We created the LGBTQ+ status variable using participant responses to three survey questions: (1) “how do you describe your gender identity?” (2) “do you identify as transgender?” and (3) “which of the following best describes your sexual orientation?” We coded youth who responded to one or more of the following as LGBTQ+: (1) gender as “non-binary”, genderfluid”, or “other”; (2) sexual orientation as “gay or lesbian”, “bisexual”, “pansexual”, “asexual”, “questioning”, “other”; or (3) “yes” to identifying as transgender. The LGBTQ+ status variable captures youth who identify as queer and/or transgender. We used a binary variable capturing LGBTQ+ status (0 = cisgender and straight, 1 = LGBTQ+) for the main independent variable of interest.

#### 2.2.3. Peer Victimization

The Dane County Youth Assessment (DCYA) questionnaire assessed peer victimization using the University of Illinois Victimization Scale [83]. Students reported the frequency (i.e., “never” to “5 or more times”) of experiencing the following in the past 30 days: (1) “someone made unwanted sexual comments to me”, (2) “I got hit and pushed by other students”, (3) “other students picked on me”, (4) “other students made fun of me”, or (5) “other students called me names”. The scores of each item were averaged to construct the peer victimization scale. We standardized the peer victimization scale to a mean of zero and used standard deviations for the analysis. Positive scores indicate greater victimization and negative scores represent fewer instances of victimization than the sample mean (α = 0.84).

#### 2.2.4. Anxiety

The Dane County Youth Assessment (DCYA) questionnaire assessed anxiety using the Generalized Anxiety Disorder (GAD)-7 [84]. Students reported the frequency of experiencing the following in the last 30 days using a four-point Likert scale ranging from “not at all” to “always”: (1) “felt nervous, anxious, or on edge”; (2) “not been able to stop or control worrying”; (3) “felt problems were piling up so high that you could not handle them”; (4) “worried too much about different things”; (5) “had trouble relaxing”; (6) “been so restless that it is hard to sit still”; (7) “become easily annoyed or irritable”; and (8) “felt afraid as if something awful might happen”. The scores of each item were averaged to construct a scale. We standardized the anxiety scale to a mean of zero and used standard deviations for the analysis, with positive scores indicating greater and negative scores representing less anxiety than the sample mean (α = 0.94).

#### 2.2.5. Suicide Attempts

The questionnaire assessed attempted suicide by asking students about the frequency of attempting suicide in the last 12 months. Students responded “no”; “yes, one time”; and “yes, more than one time.” We standardized the suicide variable to a mean of zero and used standard deviations for the analysis, with positive scores indicating greater suicide attempts and negative scores representing fewer suicide attempts than the sample mean.

#### 2.2.6. Racism/Race

We used self-reported race as a measure of identity and a proxy for racism for the main control variable. Many critical health scholars recommend conceptualizing race as a proxy for exposure to racism [85,86,87,88,89,90]. These scholars argue that racism is the key driver of racial health disparities. In a seminal piece, Jones [85] describes race as “a social construct that precisely captures the impacts of racism”. She lays out how racism contributes to health disparities via differential access to opportunities, assumptions about others’ abilities, and the internalization of stigma by race. Lett and colleagues [88] also note that measures of discrimination and racism explain only part of the observed racial health disparities. Therefore, race, as a variable, captures additional unmeasured elements of systemic racism. More recently, the American Association of Pediatrics [90] advocates for a similar approach to explicitly stating the connection between quantitative measures of race and the historical underpinnings of American racism. Because we used secondary survey data for this study, we were not able to include a more explicit measure of racism.

Students in this study reported their race as either: (1) “American Indian or Alaskan Native”, (2) “Asian (not Hmong)”, (3) “Black or African American (not Hispanic)”, (4) “Hispanic or Latino”, (5) “Asian (Hmong)”, (6) “Middle Eastern or North African”, (7) “Native Hawaiian or Pacific Islander”, (8) “White (not Hispanic)”, or (9) “Biracial or Multiracial”. Due to small sample sizes, we collapsed the following racial categories into one Other group: (1) “American Indian or Alaskan Native”, (2) “Asian (Hmong)”, (3) “Middle Eastern or North African”, and (4) “Native Hawaiian or Pacific Islander”.

### 2.3. Analytic Approach

We conducted independent *t*-tests and ordinary least squares (OLS) multivariate regression analyses to assess changes in the relationship between peer victimization, anxiety, and suicide attempts between 2018 and 2021. We restricted the analysis to items in both the middle and high school questionnaires. First, we examined whether the frequency of peer victimization, anxiety, and suicide varied between 2018 (pre-pandemic) and 2021 (mid-pandemic) using independent sample *t*-tests. We present the analysis for both the full sample and the LGBTQ+ only subsample. Second, we assessed whether the frequency of peer victimization, anxiety, and suicide varied between (1) 2018 (pre-pandemic) and 2021 (mid-pandemic) and (2) LGBTQ+ and cisgender, straight students using OLS multivariate regression analyses with controls for students’ race.

Third, we tested whether LGBTQ+ identity moderated the changes in peer victimization, anxiety, and suicide attempts from 2018 to 2021 using OLS multivariate regression analysis with controls for student race. In this model: (1) LGBTQ+ identity serves as the independent variable; (2) year, race, and the interaction between LGBTQ+ identity and year serve as covariates; and (3) peer victimization, anxiety, and suicide attempts serve as dependent variables. From this model, we capture (1) changes in peer victimization, anxiety, and suicide attempts between 2018 and 2021 among cisgender, straight youth; (2) differences in rates of peer victimization, anxiety, and suicide attempts between cisgender, straight students and LGBTQ+ students in 2018; and (3) whether LGBTQ+ identity moderated the changes in peer victimization, anxiety, and suicide attempts from 2018 to 2021. To account for the clustering of students in schools, we estimate all OLS multivariate regression models with clustered robust standard errors by school.

## 3. Results

### 3.1. Do Rates of Peer Victimization, Anxiety, and Suicide Attempts Change from 2018 to 2021 (before and during the COVID-19 Pandemic)?

We present the comparison of rates of peer victimization, anxiety, and suicide attempts before and during the pandemic in Table 2. We find statistically significantly lower rates of peer victimization (t(40,902) = 45.453, *p* = 0.000), greater attempts of suicide (t(40,902) = −10.334, *p* = 0.000), and lower anxiety (t(40,902) = 5.020, *p* = 0.000) in the 2021 cohort compared to the 2018 cohort.

The drop in reported peer victimization from 2018 to 2021 remains when we restrict the sample to only LGBTQ+ youth. However, LGBTQ+ youth reported a greater change in peer victimization (t(7184) = 25.624, *p* = 0.000) than the full sample. LGBTQ+ youth report a different trend in anxiety and suicide attempts compared to the changes in these variables found in the full sample. LGBTQ+ youth reported statistically significantly fewer attempts of suicide (t(7184) = 6.832, *p* = 0.000) but greater anxiety (t(7184) = −2.723, *p* = 0.000) in 2021 compared to 2018.

In Table 3, we present the OLS multivariate models assessing whether rates of peer victimization, anxiety, and suicide attempts vary between (1) 2018 (pre-pandemic) and 2021 (during pandemic) and (2) LGBTQ+ and cisgender, straight adolescents when controlling for students’ race. First, students in the 2021 cohort reported 0.484 $\left(\pm 0.020\right)$ and 0.095 $\left(\pm 0.015\right)$ standard deviation less peer victimization and suicide attempts than students in the 2018 cohort. We find no difference in rates of anxiety between 2018 and 2021 among all youth. Second, LGBTQ+ students reported 0.317 $\left(\pm 0.022\right)$, 0.860 $\left(\pm 0.024\right)$, and 0.407 $\left(\pm 0.020\right)$ standard deviation higher rates for peer victimization, anxiety, and suicide attempts than cisgender, straight students, respectively.

### 3.2. Does Sexual Orientation and Gender Identity Moderate the Pre- to during-COVID-19 Pandemic Changes in Peer Victimization, Anxiety, and Suicide Attempts?

The OLS multivariate models presented in Table 4 assesses whether sexual orientation and gender identity moderate the relationship between the 2018 to 2021 changes in students’ experiences of peer victimization, anxiety, and suicide attempts with controls for students’ race.

#### 3.2.1. Changes in Peer Victimization, Anxiety, and Suicide Attempts before and during the COVID-19 Pandemic among Cisgender and Straight Youth

Cisgender and straight youth reported experiencing 0.431 (±0.020) standard deviation less peer victimization in 2021 compared to 2018. We find no statistically significant difference in rates of anxiety among cisgender and straight youth between 2018 and 2021. Cisgender and straight youth in the 2021 cohort reported 0.058 (±0.013) standard deviation fewer attempts of suicide than cisgender and straight youth in the 2018 cohort. 

#### 3.2.2. Differences in Peer Victimization, Anxiety, and Suicide Attempts in 2018 between LGBTQ+ and Cisgender and Straight Youth

In 2018, LGBTQ+ youth reported experiencing higher rates of peer victimization, anxiety, and suicide attempts than their cisgender and straight peers. Compared to their cisgender and straight peers, LGBTQ+ youth experienced almost one-half a standard deviation, 0.477 (±0.013), greater peer victimization. We also find that LGBTQ+ youth experienced 0.841 (±0.027) and 0.517 (±0.038) standard deviation higher rates of anxiety and suicide attempts compared to their cisgender, straight counterparts.

#### 3.2.3. LGBTQ+ Identity Moderates Decreases in Peer Victimization and Suicide Attempts from before to during the COVID-19 pandemic

The moderation analysis suggested an even greater decrease in peer victimization and suicide attempts for LGBTQ+ students compared to cisgender, straight students from before to during the COVID-19 pandemic. Between 2018 and 2021, LGBTQ+ students experienced an additional 0.288 (±0.034) and 0.199 (±0.048) standard deviation decline in peer victimization and suicide attempts. We find no significant differences in the relationship between the COVID-19 pandemic and anxiety when we add the 2021 and LGBTQ+ status interaction term to the model.

## 4. Discussion

Our results complicate the existing literature concerning COVID-19-related school disruptions and provides additional evidence implicating school climate in LGBTQ+ students’ well-being. In our diverse sample of Midwestern middle and high schools, LGBTQ+ students in 2021 reported an even greater drop in suicide attempts and peer victimization compared to cisgender, straight students. Similarly, LGBTQ+ students reported an even steeper increase in anxiety compared to cisgender, straight students. These results suggest that the COVID-19-related school disruptions may have given LGBTQ+ students a reprieve from some social stressors and health risks, while still exacerbating anxiety. Only one qualitative study [77] has similarly examined how the COVID-19 pandemic-related disruptions had both beneficial and negative consequences for LGBTQ+ youth. This aspect of our findings points to the urgent need for policymakers and school leaders to address the systemic harms LGBTQ+ students experience as schools have shifted back to in-person schooling again.

### 4.1. Peer Victimization Decreased during the COVID-19 Pandemic for All Youth but Decreased at a Greater Rate for LGBTQ+ Students

Many scholars predicted that the COVID-19 pandemic would exacerbate existing inequities among marginalized student populations [1,2,3,4,5,7]. First, our results suggest American, in-person schooling continues to hamper LGBTQ+ students’ well-being. When students experienced COVID-19-related school disruptions, such as the move to virtual schooling, the LGBTQ+ students in this study’s schools reported fewer negative school-based social interactions during the pandemic than when all students attended in-person schooling in the pre-pandemic cohort. Both the bivariate and multivariate regression analysis with interactions for the year, sexual orientation, and gender identity indicated peer victimization decreased at a greater rate for LGBTQ+ youth compared to their cisgender, straight peers. This finding fits the relatively sparse literature concerning LGBTQ+ students’ educational experiences during the pandemic. In an analysis of online LGBTQ+ chat-based support group transcripts during the pandemic, Fish and colleagues [77] similarly reported some youth expressed “freedom from ‘transphobic and homophobic people in real life for a while’” (p. 451). Our results provide additional quantitative evidence of the prevalence of Fish et al.’s qualitative findings.

As schools shut their doors to in-person instruction in response to stay-at-home orders, students experienced less contact with their peers during the pandemic. Less physical contact with peers would account for the lower instances of peer victimization among all youth, but especially LGBTQ+ youth who disproportionately experienced peer victimization in schools prior to the suspension of in-person schooling [13,15,16,19,32]. In line with this existing research, we find that LGBTQ+ youth face greater instances of peer victimization than their cisgender and straight peers, but this gap decreased by 2021. In other words, LGBTQ+ youth reported experiencing lower rates of peer victimization during the pandemic than LGBTQ+ students prior to the pandemic, but the overall rate remained ⅙ of a standard deviation higher when compared to cisgender, straight youth during the pandemic. This persistent gap in victimization may highlight how current efforts to curb bullying in schools do not account for systemic power and oppression.

### 4.2. Anxiety Increased during the COVID-19 Pandemic for LGBTQ+ Students

Although victimization among all youth decreased during the pandemic, the pandemic further exacerbated mental health disparities among LGBTQ+ and cisgender, straight youth. Even before the pandemic, researchers documented increasing rates of anxiety among youth [91,92] and sizable differences in rates of anxiety between LGBTQ+ and cisgender and straight youth [92]. Even though scholarship has indicated that peer victimization does not fully account for poorer mental health outcomes for LGBTQ+ students [32], our results show an inverse relationship between anxiety and peer victimization during the COVID-19 pandemic. Although we did not find increased anxiety among all youth in our sample, our bivariate analyses indicated that LGBTQ+ youth experienced higher rates of anxiety symptoms during the pandemic than before the pandemic. Additionally, the OLS multivariate models suggest that LGBTQ+ youth experienced greater anxiety than their cisgender and straight peers. As students moved from in-person to virtual learning, the COVID-19 pandemic may have reduced LGBTQ+ students’ exposure to peer victimization, but out-of-school stressors may have disproportionately contributed to LGBTQ+ students’ anxiety. Some students may have lost access to supportive school environments and were forced to spend more time with unsupportive family members than before the pandemic [7,9,10,77]. Our results indicating LGBTQ+ youth experienced increased anxiety during the pandemic could reflect scholars’ concerns about pandemic-induced uncertainty, social isolation, decreased access to mental health resources in schools, unsupportive familial environments, and increased anti-LGBTQ+ rhetoric and legislation over the same time period [7,8,9,10,77].

### 4.3. Suicide Attempts Decrease during the COVID-19 Pandemic for All Youth but Decreased at a Greater Rate for LGBTQ+ Students

The increase in suicide attempts among all adolescents in our sample reflected a national trend among American adolescents, as well as recent scholarship that describes increased suicide attempts among adolescents during the COVID-19 pandemic. Yet, when we control for race, gender, and sexuality, this increase among all students was no longer significant. In a recent study using the Adolescent Behaviors and Experiences Survey (ABES) data, Jones [43] found that suicide attempts among adolescents increased from 5.8% to 11.9% during the pandemic. Using data from emergency visits concerning attempted suicides, Yard [44] similarly revealed that suicide attempts increased among 12–25-year-old Americans from 2019 to 2021. For adolescent girls, emergency visits related to suicide were up by 50%. Yet, Charpignon et al.’s [39] assessment of suicide counts from 14 state health departments describes the difference in suicide rates across states. Although most states reported increases in adolescent suicides, several states reported decreases in suicide during the COVID-19 pandemic. Although different data sources and populations make direct comparisons difficult, the bivariate results concerning suicide for all students in our sample mirror these national trends. Still, when we controlled for demographic differences, our regression analysis revealed additional variation in the characteristics of students who reported increased suicide attempts.

On the contrary, our sub-analysis of LGBTQ+ youth revealed a decrease in suicide attempts from 2018 to 2021. The results from the restricted LGBTQ+ sample contrast the increased suicide attempts among the full DCYA sample between 2018 and 2021. Even with these increases in suicide rates among all youth, LGBTQ+ suicide attempts in 2021 still remained higher than the suicide attempts among cisgender, straight youth in 2021, given LGBTQ+ students had significantly higher rates in 2018. Raifman et al. [45] reported a similar decline in suicide attempts among LGB youth over time, but still remain more likely to attempt suicide than cisgender, straight youth. However, at least two national surveys of LGBTQ+ youth suggest suicide attempts have remained stable [33,93], so our study may reflect regional or methodological differences.

Several scholars have described the direct relationship between increased bullying and peer victimization with increased suicide rates among LGBTQ+ youth [94,95,96,97]. Although victimization does not explain all of the poor health concerns among LGBTQ+ youth, the relationship between victimization and suicide attempts has been well-established [32]. Many studies describe a similar decrease in both suicide rates and victimization with the presence of greater structural supports, such as inclusive or supportive state legislation [98,99,100]. Our study is not in a state with inclusive hate crime or discrimination legislation. However, the changing in-school context associated with decreased victimization could have contributed to fewer suicide attempts among LGBTQ+ youth.

### 4.4. Limitations

The results of this study should be interpreted in light of several methodological considerations. First, we capture the COVID-19 pandemic by assessing changes between 2018 and 2021. We acknowledge there may be several factors unrelated to the pandemic that could have influenced peer victimization, anxiety, and suicide attempts among LGBTQ+ youth between 2018 and 2021. With only two time points in this analysis, regression to the mean could have also influenced our estimates. Our access to the data, differences in the wording of items on the middle and high school surveys, and availability of items about LGBTQ+ identities limited a longitudinal analysis to assess trends before 2018.

Second, the survey study changed its procedures in 2021, so some students completed the survey at home. These procedure changes resulted in some demographic differences between the 2018 and 2021 cohorts. The 2021 cohort was less racially diverse than the 2018 cohort. We used sampling weights and control for students’ race in the OLS multivariate models to adjust for some of these differences. Although sampling weights help provide better estimates of our variables of interest, the demographic variation suggests the most vulnerable students might be missing from our analysis. Thus, our results should be considered conservative estimates of changes in peer victimization, anxiety, and suicide attempts over time. Similar to national trends [101,102], the percentage of LGBTQ+ students in the sample increased from 14% in 2018 to 22% in 2021. The social and political changes contributing to an increase in students who explore and feel comfortable identifying as LGBTQ+ could also play a role in decreased victimization and suicide attempts. A deeper examination of these relationships was outside this study’s scope.

Third, the political and social context could limit the generalizability of the results. The survey study occurred in a county where 76% of the voters voted for Democratic President Joseph Biden in 2020. It is possible schools in other counties saw an increase in victimization and suicide attempts due to increased marginalization related to anti-LGBTQ+ political conversations and COVID-19-related disruptions [103].

Fourth, the use of secondary data and merging data across the middle and high school surveys limited our ability to evaluate other relevant factors, such as depression, acceptance of family members, and disclosure status. Additionally, our study focuses on changes in school climate among students captured in a county-wide survey. We were not able to capture changes in individual students or their home climates, which can be more difficult to intervene in than school environments. Similarly, the sample size limited our description of the varied student population represented in the LGBTQ+ variable or additional controls. We did not have the statistical power to conduct additional analyses that would take other identities and statuses, such as race, socioeconomic status, immigrant status, geography, or more specific gender identities and sexual orientations, in our models.

## 5. Conclusions

LGBTQ+ students have a right to a safe learning environment, and it should not take pandemic-related closures to decrease victimization and suicide attempts. Although LGBTQ+ youth can thrive and lead in K-12 schools, our results suggest the extent to which many schools continue to be harmful places for LGBTQ+ youth and the additional need to attend to the safety and well-being of LGBTQ+ students who have to attend school in person. Compared to pre-pandemic LGBTQ+ student reports, LGBTQ+ students reported less peer victimization and suicide attempts during the COVID-19 pandemic. Yet, scholars predicted that the COVID-19 pandemic interruptions in K-12 schooling would put LGBTQ+ and other marginalized youth at even greater risk of experiencing academic, emotional, and physical setbacks [3,5,7,9,10]. Our findings provide a nuanced understanding of how the move from in-person to virtual or hybrid learning during the pandemic may have provided LGBTQ+ students’ some relief from harmful in-school social interactions.

It is worth emphasizing that LGBTQ+ students still had worse anxiety, victimization, and suicide attempts during the COVID-19 pandemic compared to cisgender, straight youth. Again, one of the limitations of using existing survey data was that we were not able to examine changes for individual students, the overall total effects on LGBTQ+ students’ wellness, or the long-term impact of the pandemic. The measures for the main outcomes in this study—peer victimization, anxiety, and suicide attempts—were all global measures of health and well-being. Additionally, the analysis of missing data discussed in our methods section suggests that our results may be conservative estimates. Although we used sample weights in all of our analyses, it is possible that the youth that were most severely impacted by the pandemic may be missing from the pandemic cohort data.

Policymakers, community leaders, and parents can target the structural stressors (i.e., policies, curriculum, social interactions) that exacerbate poor health outcomes, such as anxiety and suicide attempts [38,49]. Kosciw and colleagues [22,66] have demonstrated positive associations between inclusive policy protections and lower victimization, greater staff intervention in anti-LGBTQ+ bias, and better school safety. Leaders can institute intensive anti-bullying plans that establish school-wide expectations for positive behavior, incorporate social emotional learning skills in the classroom, involve families, and build all relevant stakeholders’ skills in preventing bullying [104,105]. Additionally, LGBTQ+-inclusive curriculum is related to lower levels of bullying and higher levels of safety in schools [69]. A growing literature also points to the importance of educational leaders employing multiple strategies and engaging in school-wide continuous learning to support all students in their gender journeys [106,107,108,109]. Taken together, a multi-pronged approach could improve school climates and students’ well-being.

## Figures and Tables

**Table 1:** Demographic characteristics of participants in the analytic sample.

	2018		2021	
	N	%	N	%
	24,180	100.00%	16,724	100.00%
Gender Identity				
Cisgender Male	11,864	49.10%	7610	45.50%
Cisgender Female	11,598	48.00%	8301	49.60%
Transgender, Non-binary, Gender Fluid, Other	718	3.00%	813	4.90%
Sexual Orientation				
Straight/Heterosexual	20,899	86.40%	13,032	77.90%
Gay or Lesbian	418	1.70%	481	2.90%
Bisexual	1413	5.80%	1539	9.20%
Pansexual	203	0.80%	452	2.70%
Asexual	81	0.30%	207	1.20%
Questioning my sexual orientation	764	3.20%	1013	6.10%
Other	402	1.70%	0	0.00%
Race				
White	16,715	69.10%	12,589	75.30%
Asian (not Hmong)	1076	4.40%	723	4.30%
Black/African American	1429	5.90%	668	4.00%
Hispanic or Latino	1997	8.30%	1067	6.40%
Bi-racial/Multi-racial	2185	9.00%	1173	7.00%
Other	778	3.20%	504	3.00%

**Table 2:** Independent sample *t*-tests comparing rates of peer victimization, anxiety, and suicide before and during the COVID-19 pandemic among LGBTQ+ students.

**Full Sample**	**Combined** **(40,904)**		**2018** **(24,180)**		**2021** **(16,724)**		**Mean Difference**	
	**Mean**	**SE**	**Mean**	**SE**	**Mean**	**SE**	**t**	***p*-Value**
Peer Victimization	−0.012	0.005	0.169	0.007	−0.275	0.006	45.453	0.000 ***
Suicide Attempts	−0.008	0.005	−0.050	0.006	0.054	0.008	−10.334	0.000 ***
Anxiety	−0.012	0.005	0.008	0.007	−0.042	0.007	5.020	0.000 ***
**LGBTQ+ Sample**	**Combined** **(7186)**		**2018** **(3433)**		**2020** **(3753)**		**Mean Difference**	
	**Mean**	**SE**	**Mean**	**SE**	**Mean**	**SE**	**t**	***p*-Value**
Peer Victimization	0.225	0.014	0.594	0.024	−0.112	0.015	25.624	0.000 ***
Suicide	0.340	0.019	0.473	0.029	0.219	0.024	6.832	0.000 ***
Anxiety	0.709	0.012	0.676	0.017	0.740	0.016	−2.723	0.007 ***

Note: SE refers to standard error. *** *p* < 0.01.

**Table 3:** Multivariate OLS regression examining rates of peer victimization, anxiety, and suicide attempts before and during the COVID-19 pandemic.

	Victimization		Anxiety		Suicide	
	*b*	SE	*b*	SE	*b*	SE
2021 (vs. 2018)	−0.484 ***	0.020	0.004	0.019	−0.095 ***	0.015
LGBTQ+ (vs. Cisgender and Straight)	0.317 ***	0.022	0.860 ***	0.024	0.407 ***	0.020
Race (White omitted)						
Asian (not Hmong)	−0.108 ***	0.039	0.004	0.034	−0.035 **	0.016
Black/African American	0.045 **	0.022	−0.019	0.033	0.134 ***	0.025
Hispanic or Latino	−0.081 ***	0.024	−0.002	0.024	0.059 **	0.025
Bi-racial/Multi-racial	0.097 ***	0.017	0.116 ***	0.028	0.107 ***	0.034
Other	0.017	0.035	0.093 **	0.036	0.171 ***	0.041
Constant	0.115 ***	0.022	−0.182 ***	0.020	−0.081 ***	0.011

Note: SE refers to standard error. OLS multivariate regression models estimated with clustered robust standard errors by school. *** *p* < 0.01, ** *p* < 0.05.

**Table 4:** Multivariate OLS regression analysis examining whether sexual orientation/gender identity moderates the relationships between pre-/during-pandemic peer victimization, anxiety, and suicide attempts.

	Peer Victimization		Anxiety		Suicide	
	*b*	SE	*b*	SE	*b*	SE
2021 (vs. 2018)	−0.431 ***	0.020	−0.002	0.017	−0.058 ***	0.013
LGBTQ+ (vs. Cisgender and Straight)	0.477 ***	0.027	0.841 ***	0.027	0.517 ***	0.038
LGBTQ+ in 2021 (vs. LGBTQ+ in 2018)	−0.288 ***	0.034	0.033	0.044	−0.199 ***	0.048
Race (White omitted)						
Asian (not Hmong)	−0.111 ***	0.039	0.004	0.034	−0.038 **	0.016
Black/African American	0.046 **	0.022	−0.019	0.033	0.134 ***	0.025
Hispanic or Latino	−0.083 ***	0.024	−0.002	0.024	0.058 **	0.025
Bi-racial/Multi-racial	0.099 ***	0.017	0.116 ***	0.028	0.108 ***	0.034
Other	0.013	0.035	0.094 **	0.036	0.168 ***	0.041
Constant	0.092 ***	0.022	−0.180 ***	0.020	−0.097 ***	0.010

Note: SE refers to standard error. OLS multivariate regression models estimated with clustered robust standard errors by school. *** *p* < 0.01, ** *p* < 0.05.

## Data Availability

Not applicable.
